# Mode of induction of platelet-derived extracellular vesicles is a critical determinant of their phenotype and function

**DOI:** 10.1038/s41598-020-73005-3

**Published:** 2020-10-22

**Authors:** P. M. Ferreira, E. Bozbas, S. D. Tannetta, N. Alroqaiba, R. Zhou, J. T. B. Crawley, J. M. Gibbins, C. I. Jones, J. Ahnström, P. Yaqoob

**Affiliations:** 1 School of Chemistry, Food and Pharmacy, University of Reading, Reading, RG6 6AP UK; 2 Department of Immunology and Inflammation, Centre for Haematology, Imperial College London, London, UK; 3 Institute for Cardiovascular & Metabolic Research, School of Biological Sciences, University of Reading, Reading, UK

**Keywords:** Platelets, Predictive markers

## Abstract

Platelet-derived extracellular vesicles (PDEVs) are the most abundant amongst all types of EVs in the circulation. However, the mechanisms leading to PDEVs release, their role in coagulation and phenotypic composition are poorly understood. PDEVs from washed platelets were generated using different stimuli and were characterised using nanoparticle tracking analysis. Procoagulant properties were evaluated by fluorescence flow cytometry and calibrated automated thrombography. EVs from plasma were isolated and concentrated using a novel protocol involving a combination of size exclusion chromatography and differential centrifugation, which produces pure and concentrated EVs. Agonist stimulation enhanced PDEV release, but did not alter the average size of EVs compared to those produced by unstimulated platelets. Agonist stimulation led to lower negatively-charged phospholipid externalization in PDEVs, which was reflected in the lower procoagulant activity compared to those generated without agonist stimulation. Circulating EVs did not have externalized negatively-charged phospholipids. None of the 4 types of EVs presented tissue factor. The mechanism by which PDEV formation is induced is a critical determinant of its phenotype and function. Importantly, we have developed methods to obtain clean, concentrated and functional EVs derived from platelet-free plasma and washed platelets, which can be used to provide novel insight into their biological functions.

## Introduction

Circulating extracellular vesicles (EVs) originate from a number of sources, including platelets, erythrocytes, leukocytes and endothelial cells^1^. Recent data suggests their involvement in a range of physiological and pathophysiological processes, including inflammation, cell communication, coagulation and metastasis^2,3^. EVs are commonly classified into exosomes (30–150 nm), microvesicles (100–1000 nm; also referred to as microparticles or ectosomes), and apoptotic bodies (1000–3000 nm)^4^. However, definitive discrimination has not yet been established due to the overlapping sizes and lack of accurate methods for separation and characterization. Many studies therefore use the generic term, EVs, together with a description of the size range of the particles under investigation^5^. The most abundant type of EVs in circulation are platelet-derived extracellular vesicles (PDEVs)^6^. Originally identified in platelet-free plasma, PDEVs were shown to be rich in negatively-charged phospholipids and demonstrated to support coagulation^7^. Studies have shown PDEVs to be released upon platelet activation by agonists, hemodynamic stress, ageing and inflammatory pathologies^8^ and while studied widely in vitro there is little understanding of the degree to which the PDEVs generated ex vivo are similar to those in the circulation, both in terms of composition and of functional properties.

Phospholipids in plasma membranes are organized in two layers, with phosphatidylcholine (PC) in the outer leaflet facing the circulation, and phosphatidylserine (PS) and phosphatidylethanolamine (PE) facing the inner leaflet. During the normal haemostatic response, platelets can become activated and PS and PE are relocated to the outer leaflet, creating a negatively-charged membrane surface which supports the formation of protein complexes that together form the coagulation cascade. This chain of enzymatic reactions leads to the generation of thrombin, followed by fibrin and ultimately, the formation of a stable platelet plug^9^. Negatively-charged PDEVs can serve as additional surfaces where coagulation cascade complexes can assemble^10^. Clinical evidence suggests that PDEVs could protect from bleeding during idiopathic thrombocytopenic purpura, while their dramatic release could be related to neurological complications of thrombotic origin^11^. Importantly, PDEVs have shown to be highly heterogenous in size and composition^12^ and not all PDEVs externalize PS/PE^13^, but the mechanisms leading to their exposure and functional activity of these different populations are poorly understood.

The structural diversity and dimensions of PDEVs produced by quiescent vs agonist-stimulated platelets are different^14^. However, whether vesicles produced by different stimuli also have distinctive functions, particularly procoagulant function, is not known. In the current study we have significantly advanced earlier observations by evaluating novel methods for PDEV generation and by demonstrating that agonist stimulation of platelets from healthy volunteers led to lower phospholipid externalization in the generated PDEVs. This was reflected in the lower procoagulant function of these PDEVs. In contrast, circulating EVs isolated from healthy volunteers did not have externalized negatively charged phospholipids and did not present tissue factor.

## Methods

### Subject recruitment and characteristics

Male and female subjects aged 18–60 years and free of diagnosed disease were recruited to provide blood samples. All procedures were conducted according to the principles of the Declaration of Helsinki and approved by the School of Chemistry, Food and Pharmacy Research Ethics committee (project 15/17), on behalf of the University of Reading Ethics Committee. All subjects gave written informed consent before providing samples.

### Blood collection and platelet isolation

Blood from fasted volunteers was obtained by venepuncture from the median cubital vein using a 19G butterfly needle. The first 5 ml of collected blood was discarded. The blood to be used in this study was drawn into a vacutainer containing tri-sodium citrate (3.2%; Greiner Bio-One, UK) and processed within 30 min. To obtain platelet free plasma (PFP), blood was centrifuged at 1500×*g* for 15 min and the upper two thirds were collected and centrifuged again at 13,000×*g* for 2 min. The upper three quarters from each tube were collected and identified as PFP.

Blood was centrifuged at 175×*g* for 15 min to obtain platelet-rich plasma (PRP). Platelets were then isolated by further centrifugation (1000×*g*, 10 min) in the presence of prostacyclin (PGI_2_, 1 µg/ml; Sigma, UK). The resulting pellet was washed in modified Tyrode’s (MTH) buffer (containing 134 mM NaCl, 2.9 mM KCl, 0.34 mM Na_2_HPO4, 12 mM NaHCO_3_ and 1 mM MgCl_2_; pH 7.4) containing HEPES (20 mM; Sigma, UK) and re-suspended in an equal volume of MTH. Platelets were counted and adjusted to a final working concentration of 3 × 10^8^ platelets/ml.

### Purification and concentration of circulating EVs from PFP

Size exclusion chromatography (SEC), using ready made columns (qEV, Izon Science) was used for EV isolation from PFP. After equilibrating the column with 20 ml of 20 mM Tris–HCl pH 7.5, 0.15 M NaCl (TBS), 0.5 ml of PFP was layered on top and allowed to enter the column. The top reservoir was then filled with TBS and 0.5 ml fractions were collected. Protein content on fractions was measured with the Pierce BCA Protein Assay Kit (Thermo Scientific, Cat #23225). Fractions were numbered in order of collection and the EV-rich fractions free of plasma proteins (Fractions 7–10) were kept at 4 °C (Supplemental Fig. S1). This procedure was repeated 4 more times, so that EVs were isolated from 2.5 ml of PFP from each volunteer. Altogether, 20 eV rich fractions were obtained for each volunteer, pooled and centrifuged at 15,000×*g* at 4 °C for 30 min. The supernatant was removed and the pellet rich in EVs was resuspended in a final volume of 0.4 ml. These were called PFP EVs.

### Production of PDEVs from washed platelets and PRP

In the presence of 2 mM CaCl_2_, washed platelets were stimulated with either 30 µM thrombin receptor activator peptide (TRAP-6; Bachem, UK) or MTH (non-stimulated) and incubated at 37 °C for 2 h (Supplemental Fig. S2), as described previously^15^. Platelets were then removed by two sequential centrifugations at 1200×*g* for 10 min. The supernatant, containing the EVs, was collected and centrifuged at 15,000×*g* for 30 min at 4 °C. The pellet containing EVs was stored at − 80 °C. These were called TRAP-6 PDEVs and Buffer PDEVs, respectively.

PRP was stimulated with TRAP-6 using the same protocol as described for washed platelets above. Thereafter, the stimulated PRP was centrifuged to generate PFP and EVs were isolated and concentrated using the methods outlined above. These generated vesicles were called PRP+ TRAP-6 EVs.

### Calibrated automated thrombography

The procoagulant properties of the different EV preparations were assessed by calibrated automated thrombography (CAT) using a Fluoroskan Ascent FL plate reader (Thermo Labsystem) in combination with Thrombinoscope software (Synapse BV). For this purpose, a plasma pool from 13 healthy volunteers was prepared. The blood was collected in 10 mM sodium citrate and 18 µg of corn trypsin inhibitor (Enzyme Research Laboratories) per ml of blood and spun as described previously^16^.

Thrombin generation was initiated in plasma by 16.6 mM CaCl_2_ in the presence or absence of 1 or 4 pM tissue factor (TF; Dade Innovin) and/or 4 µM phospholipid vesicles. The amount of thrombin formed was monitored using 0.42 mM of Z-Gly-Gly-Arg-AMC (Bachem) and quantified against a “calibrator” sample with known amount of alpha2M-thrombin complex run in parallel with the other samples, according to standard procedures (Stago)^17^.

The EVs were quantified by absorption at 280 nm using an extinction coefficient (E1%, 1 cm) of 10 and were added to the reaction at 0–16 µg/ml.

Phospholipids 1,2-dioleoyl-sn-glycero-3-phosphocholine (DOPC), 1,2-dioleoyl-sn-glycero-3-phosphoserine (DOPS), and 1,2-dioleoyl-sn-glycero-3-phosphoethanolamine (DOPE) (Avanti Polar Lipids) were mixed at a ratio of 60:20:20 and prepared by extrusion as described previously^16,18^.

### Antibody labelling

Extracellular vesicles were assessed for their ability to bind cell marker-specific monoclonal antibody anti CD41 conjugated to Phycoerythrin (PE) (BioCytex Cat# 5112-PE100T) to detect PDEVs, Annexin V conjugated to Allophycocyannin (APC) (ThermoFisher, Cat# BMS306APC-100), which binds externalized PS residues, and duramycin (DM) (Sigma, Cat# D3168), which was conjugated to Atto-488 dye (ThermoFisher, Cat # 710369) and specifically binds PE^19^. Samples were double stained: 1–5 μl of each EV sample with both anti CD41 and Annexin V or DM was carried out for at least 30 min in the dark at room temperature, followed by dilution with Annexin V-binding buffer (10 mM HEPES, 140 mM NaCl, 2.5 mM CaCl_2_, pH 7.4).

### Flow cytometric analysis

Firstly, the limits of detection of the flow cytometer (Canto II Flow Cytometer (BD Biosciences, UK)) and the EV gate were established using standard size calibration beads (Apogee Flow Systems, Hemel Hempstead, UK), comprised of 180 nm, 240 nm, 300 nm, 590 nm, 880 nm and 1300 nm silica beads and 110 nm and 500 nm green fluorescent latex beads, to determine the optimum side scatter (SSC) and forward scatter (FSC) voltages for EV detection: 540 and 500 respectively. The EV gate included all particles of size 240 nm–1 µm, in which the lower detection limit was set by 240 nm silica beads to exclude noise, while the upper detection limit was set just above 880 nm silica beads to exclude platelets, larger debris and contaminations (Supplemental Fig. S3). Each sample was measured successively using different triggering signals. Firstly the SSC signal was used to trigger detection of EVs based on light scattering (Threshold 700) and to establish an optimum dilution, which was kept at around 300 events/second. After that, the instrument was switched to fluorescence mode and the signal was triggered on the detector correspondent to the fluorophore of the antibody of interest: CD-41 PE (λex. = 496 nm, λem. = 578 nm; Voltage 470, Threshold 350), Annexin V APC (λex. = 650 nm, λem. = 660 nm; Voltage 642, Threshold 550) and Duramycin FITC (λex. = 490 nm, λem. = 525 nm; Voltage 642, Threshold 550). Fluorescence triggering provides higher sensitivity by removing interference from noise and particles that would be detectable using light scattering triggering^13^. All chemicals were filtered using 100 nm syringe filters (Sartorius, UK) and 100 nm centrifugal devices (Sartorius, UK) and analysed for 1 min (10 µl) to assess the level of background particles that fell in the EV gate.

Events were collected for 1 min (10 µl) on the threshold corresponding to the labelled fluorophore at “Low” flow rate (10 µl/min). For anti CD41, sample incubation with its isotype control had lower fluorescence than the antibody on its own; therefore the background was set on the fluorescence produced by the antibody only.

EV positive events were calculated, by subtracting the number of events acquired from the antibody mix on each experimental day from the absolute count of positive events of the labelled sample (Supplemental Fig. S4). Ratios between phospholipid externalization (PS and PE) were calculated by dividing the number of positive events obtained on APC (PS) and FITC (PE) channels by the number of positive events obtained on the PE (CD41) channel. Data was captured using FACSDiva Software version 6.1.3 and analysed using FlowJo version 10.

### Nanoparticle tracking analysis

Size distribution and concentration of EVs were analysed by Nanoparticle Tracking Analysis (NTA) using a NanoSight NS300 instrument (Malvern Instruments). Samples were diluted in PBS to achieve a concentration of approximately 1–9 × 10^8^ particles/ml and injected into the NanoSight sample chamber using a 1 ml syringe and syringe pump. Five 1-min videos were captured at camera level 13 and frame rate of 25 per second and analysed by Nano 3.2 software.

### Statistical analysis

Statistical analyses were performed using GraphPad Prism software. Paired student’s T-test was performed for head-to-head comparisons. Non-linear fit models were generated for CAT concentration–response curves and extra sum-of-squares F test was used for comparison between curves. For all tests, p < 0.05 was considered statistically significant.

## Results and discussion

**Figure 1:**
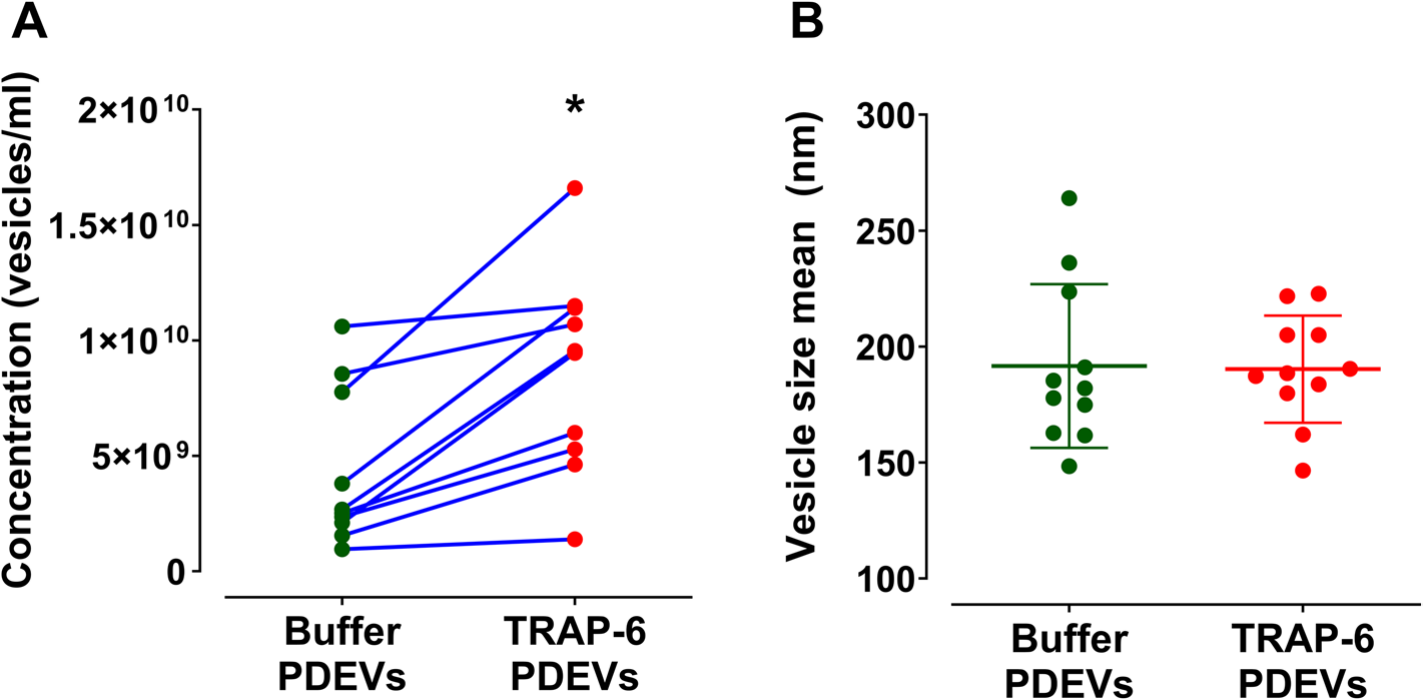
Platelet agonist stimulation facilitates the formation of EVs with no difference in average vesicle size according to NTA. (**A**) Head to head comparison of tracked vesicle concentration generated from either unstimulated (Buffer) platelets or platelets stimulated by the addition of TRAP-6. Vesicles generated from 1 ml of 3 × 10^8^ platelets in 1 ml of MTH buffer. Platelets were then removed and the supernatant injected on NTA. Two-tailed paired t-test, p = 0.001, n = 11. (**B**) Comparison of tracked vesicle average size. No significant difference was observed from both types of vesicles: Buffer PDEVs = 191.6 nm × TRAP-6 PDEVs = 190.3 nm. Results are presented as Mean ± SD, n = 8. Two-tailed paired t-test p = 0.663, n = 11.

In the current study, platelets were washed and PDEVs were generated in the presence or absence of TRAP-6 stimulation, followed by isolation under the same conditions through differential centrifugation. The generated vesicles were initially characterised by number and size using NTA. TRAP-6 stimulation enhanced PDEV release (8.6 × 10^9^ vs 4.2 × 10^9^ vesicles/ml from buffer only (p = 0.01); Fig. 1A. In contrast, there was no difference in average size (TRAP-6 PDEVs = 190.3 nm vs Buffer PDEVs = 191.6 nm; Fig. 1B. These findings are in line with previous reports where it has been demonstrated that platelet thrombin receptor stimulation boosts formation of small PDEVs (50–100 nm)^14^, whilst the average size of the thrombin-stimulated platelet-derived EVs is not statistically different from EVs generated from unstimulated platelets^12^ (Fig. 1).

The PDEV concentration steps performed, although done on a benchtop centrifuge, produce a sample which is 8.5 times more concentrated than its respective supernatant (Supplemental Fig. S5). This is imperative for the CAT assays where a sample needs to be over 100 µg/ml in protein concentration for adequate testing. The neat supernatant does not reach this concentration so we could not test its functional properties. Flow cytometry also would not be a reliable approach for characterisation given that the average size of the EVs present in the supernatant is lower than the detection limit of the settings established, therefore the choice to work with a concentrated sample.

**Figure 2:**
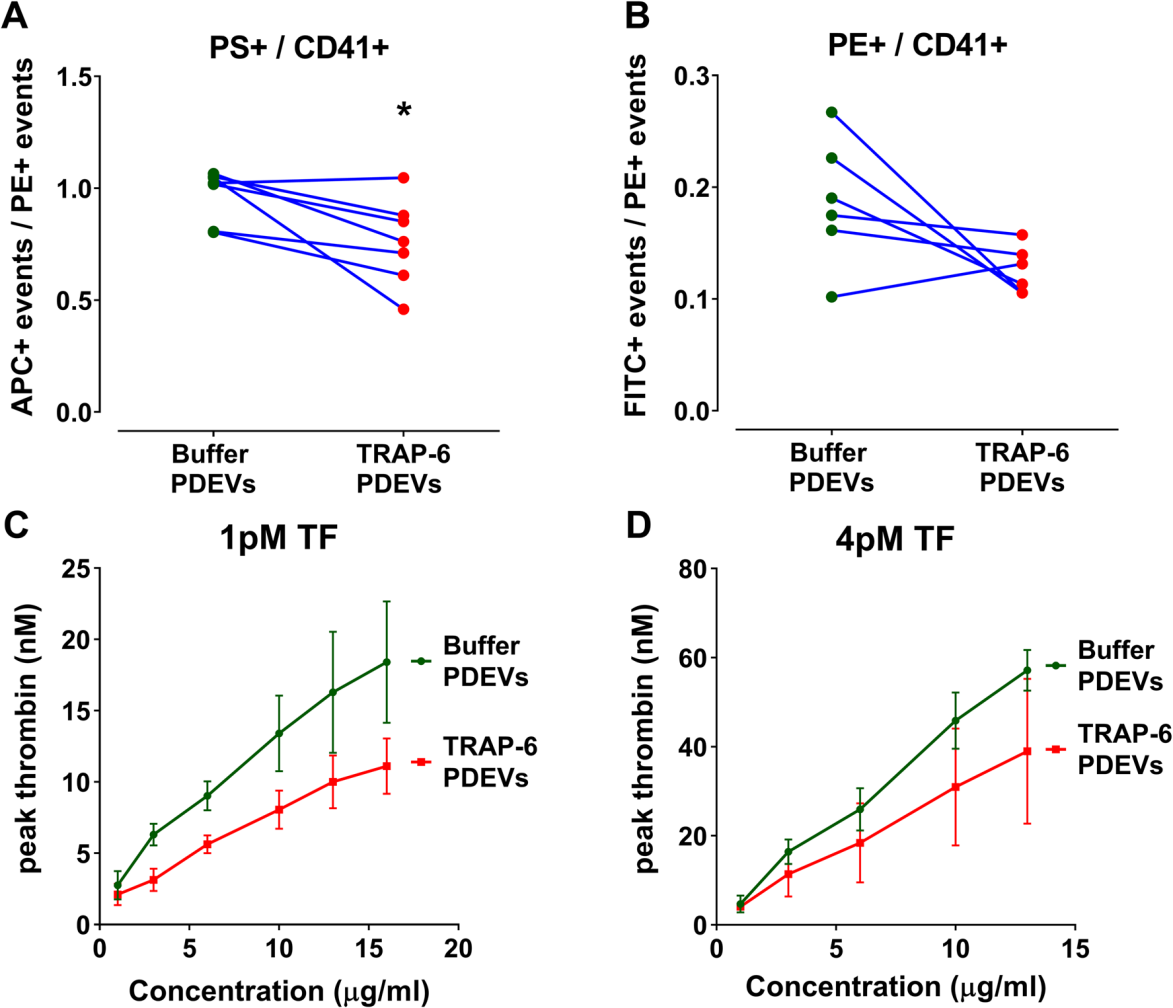
Platelet-derived EVs generated without agonist stimulation are more procoagulant than those generated by of TRAP-6. (**A**,**B**) Relationship of phosphatidylserine (PS) positive events (**A**) and phosphatidylethanolamine (PE) positive events (**B**) divided by CD41 positive events for unstimulated and TRAP-6 stimulated PDEVs. Events were captured on the fluorescence mode on the PE channel (CD41+), APC channel (PS+) and on the FITC channel (PE +). n = 6–7. Two-tailed paired t-test (**A**) p = 0.026, (**B**) p = 0.088. (**C**,**D**) Procoagulant properties of 0–16 or 0–13 µg/ml PDEVs were assessed using CAT in the presence of 1 pM (**C**) or 4 pM (**D**) TF, respectively. Dose–response curves representing peak thrombin generation are plotted against PDEVs concentration. Results are presented as mean ± SEM, n ≥ 3. A non-linear fit regression (4 parameters) was performed on (**C**,**D**) and the best-fit values were compared. (**C**) p = 0.001, (**D**) p = 0.293.

To assess whether mode of platelet stimulation influences PS and PE exposure, the vesicles were characterised using flow cytometry. TRAP-6 stimulation led to lower PS and PE externalization compared to vesicles generated in the absence of agonist (Fig. 2A,B). This was also reflected in the lower procoagulant properties of TRAP-6 stimulated PDEVs when investigated in CAT assays in the presence of TF (Fig. 2C,D).

In previous studies investigating the ability of PDEVs to support thrombin generation, it was shown that size altered their procoagulant properties where smaller vesicles (142 ± 12 nm) stimulated less thrombin generation than a larger population (173 ± 15 nm)^4,20^. The current study demonstrates that not only size is important for the negatively-charged phospholipid exposure profiles and procoagulant activity, but the stimuli that triggered PDEVs generation.

The majority of circulating EVs do not present PS on their outer surface^13^ and these are mainly PDEVs^6^. Purification methods of circulating EVs rely solely on differential centrifugation from PFP, leaving the final extract contaminated with plasma proteins and immunocomplexes^21^. These can potentially interfere with functional assays and lead to underestimation of the EV concentration if determined by protein content. Counting EVs by flow cytometry or NTA would also lead to misinterpretation, since there is no valid general marker for EVs and NTA is unable to differentiate vesicles from protein aggregates.

As a result of these limitations, we have developed a novel purification method, which produced circulating EVs free of most plasma proteins and at a concentration that allows them to be employed in functional experiments. EVs were isolated from plasma by SEC and subsequently concentrated through differential centrifugation. It has been shown previously that SEC removes 99% of soluble plasma proteins and > 95% of HDL from the purest fraction of EVs^22^, without inducing aggregation of EVs^23^ while retaining their integrity and biological activity^24^. Only Fractions 7–10 were collected in order to avoid the presence of plasma proteins. As shown on Supplemental Fig. S1, plasma proteins start to increase substantially after Fraction 11. Even if a small plasma protein contamination is unavoidable, these should remain in the supernatant after the final centrifugation step. The fact that PRP+ TRAP-6 EVs had a similar potency to TRAP-6 EVs on CAT (compare Fig. 2C, 3A) validates the novel EV SEC purification protocol from plasma since their concentration was determined by protein content, although generated via two different protocols. If there would be significant protein contamination on PRP+ TRAP-6 EVs these would have lower potency compared to TRAP-6 PDEVs.

**Figure 3:**
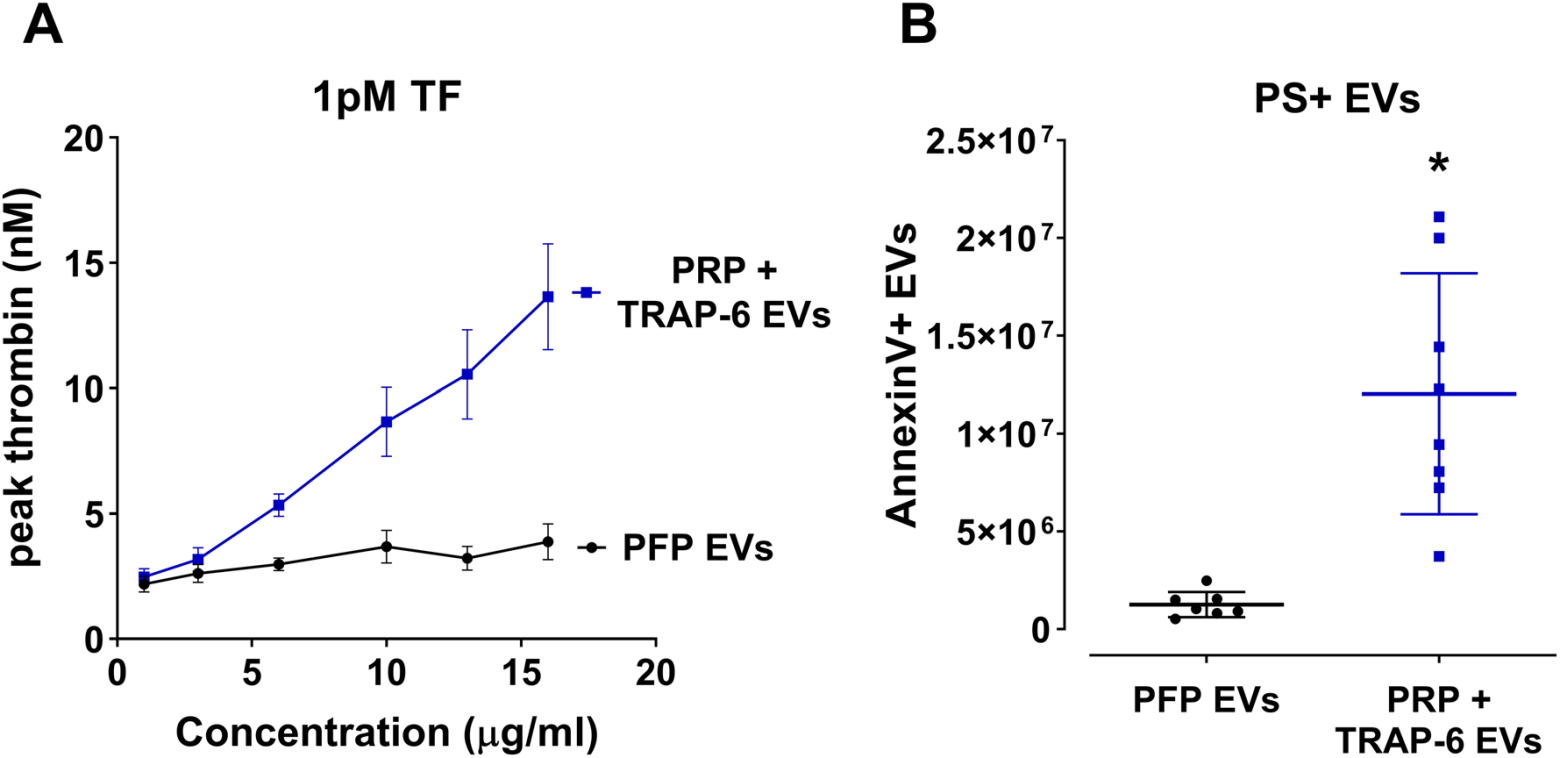
Circulating extracellular vesicles (EVs) do not externalize PS and consequently do not support thrombin generation. (**A**) The procoagulant properties of 0–16 µg/ml circulating EVs (PFP) or stimulated PDEVs+ circulating EVs (PRP) were assessed using CAT in the presence of 1 pM TF. Dose–response curves representing peak thrombin generation are plotted against vesicle concentration. Results presented as Mean ± SD, n = 4. (**B**) Phosphatidylserine exposure was determined by Annexin V binding to PFP and PRP EVs and quantified through fluorescence flow cytometry. Due to low Annexin V binding, PFP EVs do not carry negatively charged phospholipids and consequently cannot support thrombin generation on its surface. Results presented as Mean ± SD, n = 8.

To investigate how PDEVs functionally relate to circulating EVs in healthy volunteers, PRP was stimulated with TRAP-6, centrifuged to produce PFP, which was then subjected to SEC to purify the EVs. In parallel, another sample of PFP, from the same volunteer, was used to purify EVs present without any stimulation—representing the circulating EVs. The two types of EVs were isolated and quantified in exactly the same way. PDEVs isolated from ‘stimulated’ PRP supported thrombin generation in a dose-dependent manner, demonstrating that the agonist-stimulation of PRP followed by EV purification produced functional EVs (Fig. 3A). In contrast, circulating EVs (derived from unstimulated PFP) did not support thrombin generation at concentrations up to 16 µg/ml (Fig. 3A). This was explained by the low levels of PS exposure detected on the outer layer of circulating EVs, which is essential for thrombin generation (Fig. 3B).

**Figure 4:**
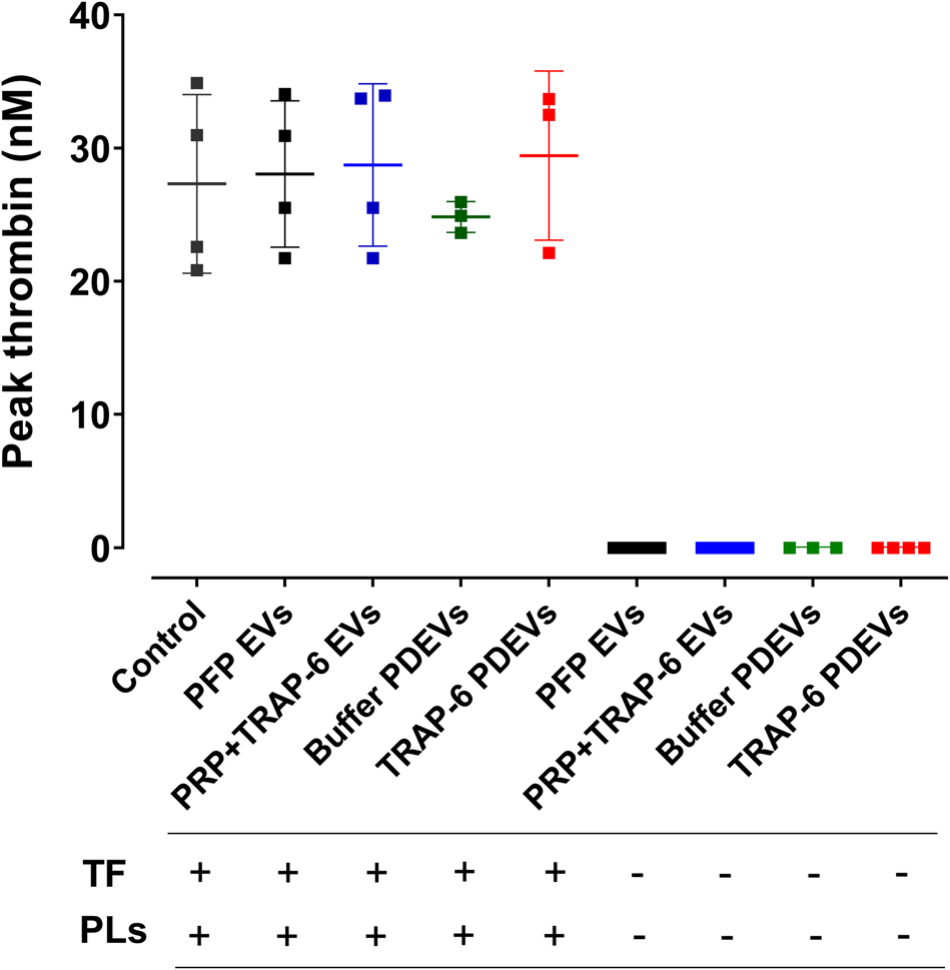
Circulating and PDEVs do not express TF. To assess whether EVs from different sources could stimulate thrombin generation in the absence of TF, 10 µg/ml of all types of EVs studied here were added to normal plasma, in the presence and absence of synthetic phospholipid membranes (4 µM). The peak thrombin generation was compared to that generated by normal plasma alone in the presence of 4 µM phospholipids and 1 pM TF. Results presented as Mean ± SD, n ≥ 3.

The presence of TF on circulating EVs and on PDEVs and its potential influence on their procoagulant function has been a matter of debate^4,25,26^. To address this, the ability of EVs (10 µg/ml) to support thrombin generation in the absence of TF, but in the presence of synthetic negatively-charged phospholipid membranes was evaluated. These results demonstrated a complete absence of thrombin generation in the absence of TF (Fig. 4).

## Conclusions

In summary, this study demonstrates that the mechanism by which PDEV formation is induced is a critical determinant of their phenotype, but not size. PDEVs derived via different mechanisms (i.e. from washed platelets vs PFP and unstimulated vs stimulated) exhibited different procoagulant properties. Novel methods were developed to obtain clean, concentrated and functional EVs derived from both PFP and from washed platelets, which can be used to provide new insights into their biological composition and function. Further studies are necessary to determine the mechanisms by which platelets release different EV populations.

## Supplementary information

Supplementary information.
