# Supplementary material for: Mode of induction of platelet-derived extracellular vesicles is a critical determinant of their phenotype and function
Source: Sci Rep. 2020 Oct 22;10:18061. doi: 10.1038/s41598-020-73005-3 (PMC7582134; doi:10.1038/s41598-020-73005-3)

List of authors:

Ferreira, PM; Bozbas, E; Tannetta, SD; Alroqaiba, N; Zhou, R; Crawley, JTB; Gibbins, JM; Jones, CI, Ahnström, J; Yaqoob, P.

Mode of induction of platelet-derived extracellular vesicles is a critical determinant of their phenotype and function

Supplemental Figure S1 – Fractions 7 to 10 have a high concentration of vesicles without plasma protein contamination. Fraction by fraction diagram of circulating extracellular vesicles purified by size-exclusion chromatography showing vesicle count on the left y-axis and protein concentration on the right y-axis. Vesicle count was performed through Nanoparticle Tracking Analysis and protein content was determined through the bicinchoninic protein assay (BCA).

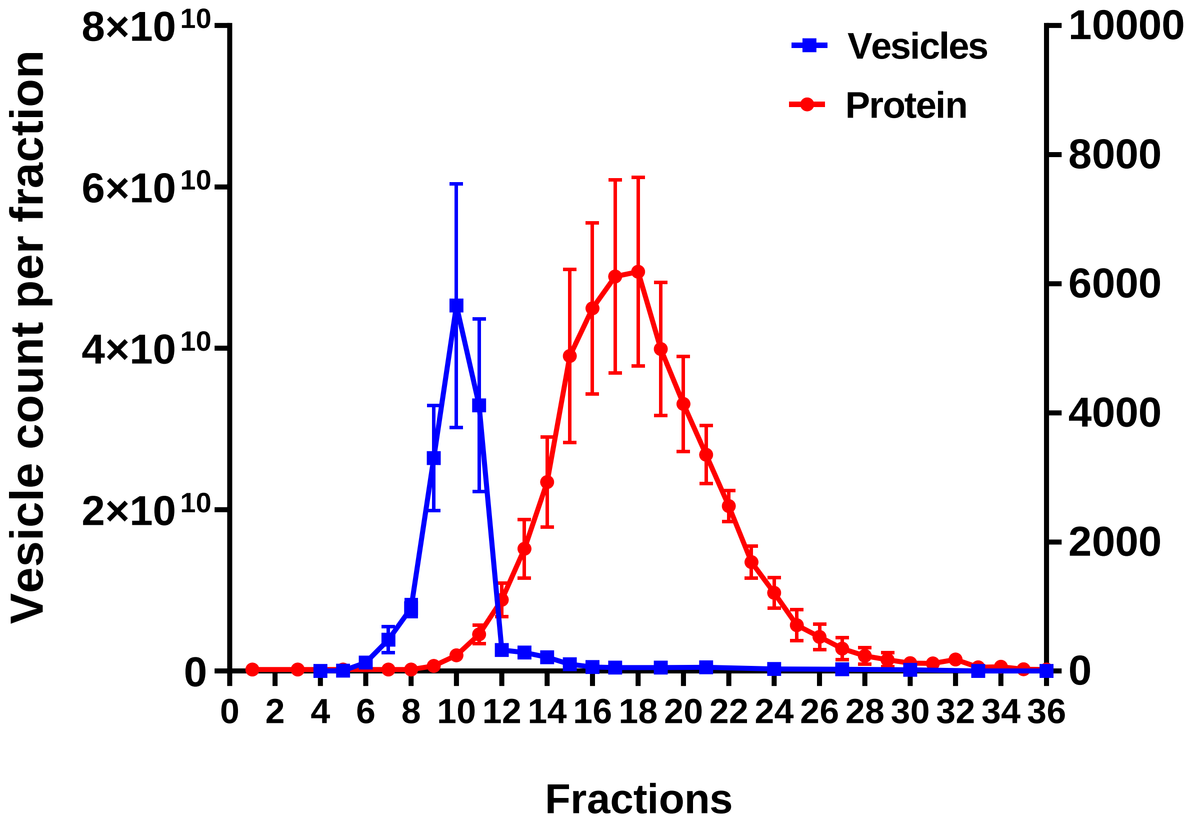

Supplemental Figure S2 – Time-course analysis of flow cytometric detection of platelet-derived extracellular vesicles. Platelets were stimulated with TRAP-6 and removed after different time-points. The neat PDEV extract was labelled with CD41 and analysed on fluorescence flow cytometry. Platelet derived extracellular vesicles (PDEVs) peaked detection after 120 minutes of stimulation.

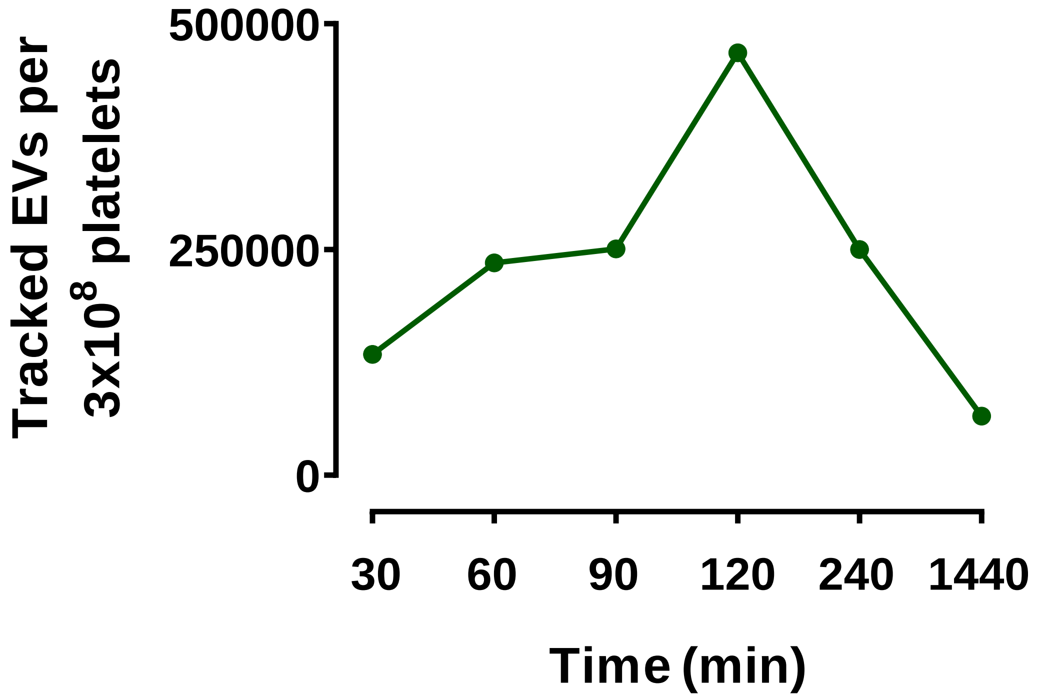

Supplemental Figure S3 – FACSCanto II flow cytometer set up. Forward scatter (FSC) vs side scatter (SSC) plot to show the EV analysis gate established by ApogeeMix beads. The gate included all particles of size 240nm-1µm, in which the lower detection limit was set by 240 nm silica beads to exclude noise, while the upper detection limit was set just above 880 nm silica beads to exclude larger debris and contaminations. SSC Voltage = 540, FSC Voltage = 500, signal triggered on SSC (Threshold 700).

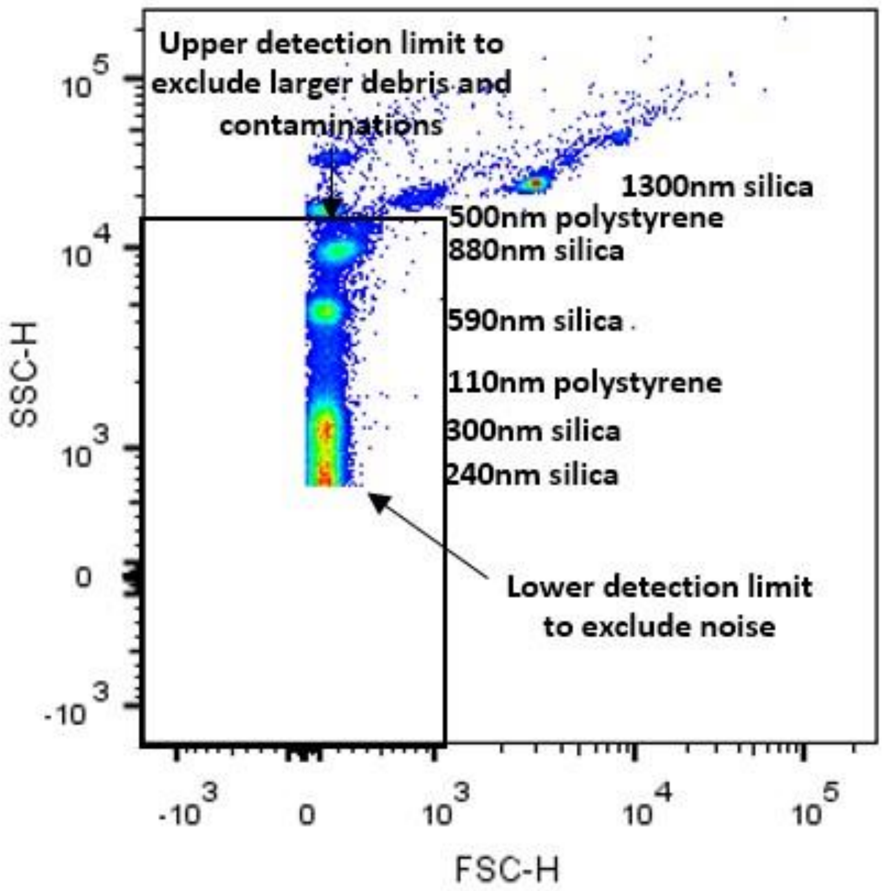

Supplemental Figure S4 – Representative flow cytometry histograms with signal triggered in fluorescence mode showing mean fluorescence intensity (MFI) of the antibody background (red) and platelet-derived extracellular vesicles (PDEVs) positive events (blue). PE = CD41, APC = Annexin V, FITC = Duramycin. The number of positive events was calculated subtracting the events collected in blue minus events collected in red.

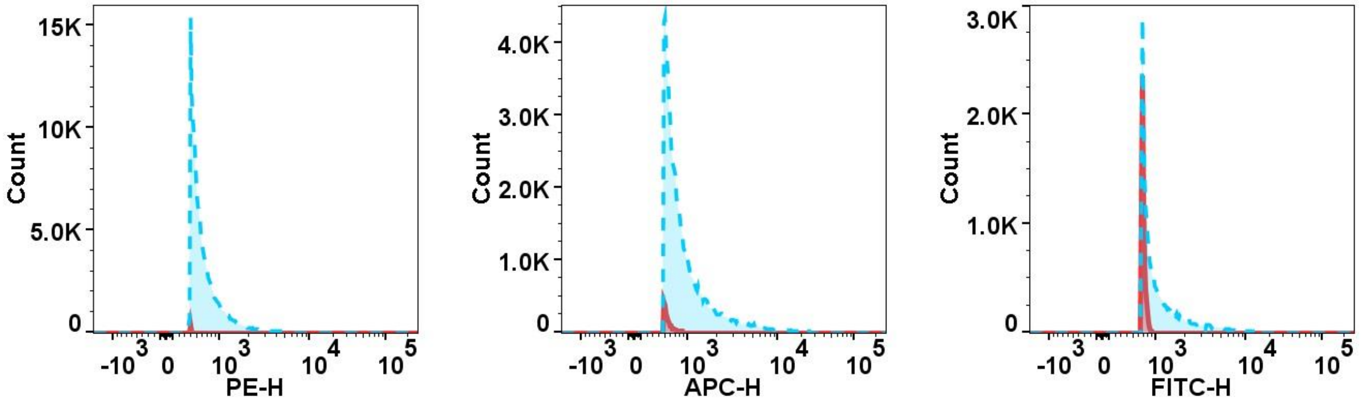

Supplemental Figure S5 – Concentration and average size from concentrated platelet-derived extracellular vesicles (PDEVs) and their respective supernatant. Washed platelets were stimulated with TRAP-6 30 µM for 2 hours at 37ºC. Platelets were then removed by two sequential centrifugations at 1,200g for 10 minutes. The supernatant, containing the EVs, was collected and centrifuged at 15,000g for 30 minutes at 4°C. The pellet containing concentrated EVs (PDEVs) and the supernatant were separated and their size and concentration was determined through Nanoparticle Tracking Analysis. The PDEVs are 8.5 times more concentrated than the supernatant. Results presented as Mean ± SEM, n=6. Two-tailed paired t-test $p < 0.05$ for both Concentration and Average size.

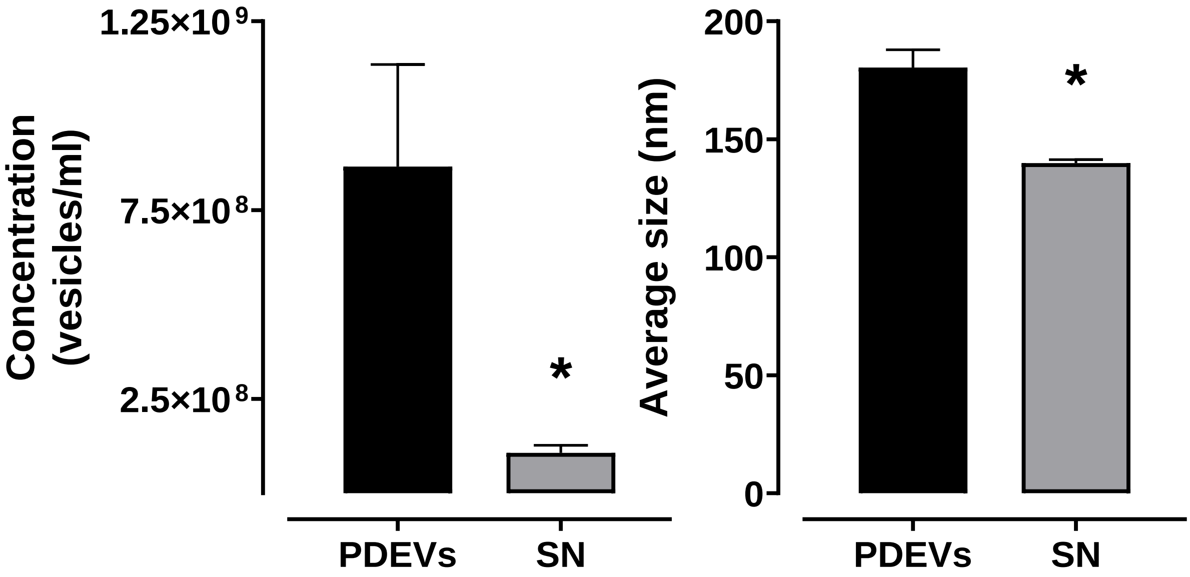

Supplement: Supplementary file 1 — Supplementary information. [file 41598_2020_73005_MOESM1_ESM.pdf]
